# A description of feeding and swallowing in neonates with hypoxic ischemic encephalopathy

**DOI:** 10.4102/sajcd.v72i2.1121

**Published:** 2025-11-20

**Authors:** Samantha Branfield, Natasha R. Rhoda, Janine Joemat, Vivienne Norman

**Affiliations:** 1 Division of Communication Sciences and Disorders, Faculty of Health Sciences, University of Cape Town, Cape Town, South Africa; 2 Department of Paediatrics and Child Health, Faculty of Health Sciences, University of Cape Town, Cape Town, South Africa; 3 Mowbray Maternity Hospital, Department of Health and Wellness, Western Cape Government, Cape Town, South Africa

**Keywords:** intrapartum hypoxia, hypoxic ischemic encephalopathy, neonate, feeding, swallowing, dysphagia, oropharyngeal dysphagia, low- and middle-income country

## Abstract

**Background:**

Intrapartum-related complications, including intrapartum hypoxia and subsequent hypoxic ischemic encephalopathy (HIE), are the second leading cause of neonatal deaths in South Africa. Complications may be associated with a substantial burden of impairment, including dysphagia.

**Objectives:**

To describe the feeding and swallowing profile of neonates with HIE in a neonatal unit in Cape Town, South Africa.

**Method:**

A descriptive, exploratory, longitudinal study of 52 participants with varying severities of HIE is presented. Clinical feeding and swallowing assessments were conducted with 13 prospective participants using the Neonatal Feeding Assessment Scale, and medical folder reviews were conducted for 39 retrospective participants. Data were analysed using descriptive and inferential statistics.

**Results:**

A median of 4 days (*p* = 0.036) to oral feeding readiness and 5 days (*p* = 0.016) to full oral feeds was observed. Participants with a severely abnormal initial amplitude-integrated electroencephalography who did not receive hypothermia treatment demonstrated significantly longer times for both outcomes. Participants across all severities presented with feeding and swallowing difficulties, primarily in the oral phase. Most participants (96.2%) were discharged on full oral feeds, while the remaining 3.8% were discharged on nasogastric tube feeds while awaiting gastrostomy placement.

**Conclusion:**

Regardless of severity, neonates with HIE are at increased risk of feeding and swallowing difficulties. Thus, early identification and management before discharge from the neonatal unit, and long-term follow-up of infants with HIE, are recommended.

**Contribution:**

This study contributes to the small body of research on feeding and swallowing difficulties in neonates with HIE and may guide future research.

## Introduction

Hypoxic ischemic encephalopathy (HIE) is one of the most serious birth complications that affect full-term neonates, and despite medical advances such as therapeutic hypothermia, it remains a major cause of life-long disability and mortality worldwide (Cetinkaya, 2024). In South Africa (SA), intrapartum-related complications, including intrapartum hypoxia and subsequent HIE, are reported as the second most prevalent causes of neonatal deaths (Masaba & Mmusi-Phetoe, 2020). The reported incidence of HIE in SA ranges from 1.5 to 13.3 per 1000 live births (Bruckmann & Velaphi, 2015; Horn et al., 2013; Lambey Nakwa et al., 2023), similar to the range of 1.5–20.3 reported in other low- and middle-income countries (LMICs) (Kukka et al., 2022) and compared with the 1.5 per 1000 live births in high-income countries (Acun et al., 2022).

Factors such as a high burden of disease, widespread poverty, limited healthcare resources and facilities, inadequate ambulance services and a shortage of healthcare workers, typical of LMICs, predispose SA to higher prevalence rates of HIE (Ballot et al., 2020). Limited access to therapeutic hypothermia treatment in SA – the standard of care for neonates with moderate to severe HIE – further compounds the risk of poor neurodevelopmental outcomes in affected neonates (Ballot et al., 2021; Kukka et al., 2022). These challenges not only contribute to the incidence of HIE but may also lead to adverse long-term outcomes (Tagin et al., 2012), further straining the healthcare system of SA.

While it is universally agreed that HIE is an important cause of neonatal mortality, for survivors, it is associated with a substantial burden of impairment (Ballot et al., 2020; Krüger et al., 2017, 2019; Malan et al., 2022). Although dependent on the extent of the cerebral injury, neonates with HIE are likely to present with varying degrees of encephalopathy, including depressed levels of consciousness, difficulty initiating and maintaining respiration, hypotonia, seizures and weakened reflexes, including delayed or absent feeding and swallowing responses (Gillam-Krakauer & Gowen, 2022). Thus, HIE has been referred to as a potential neurological cause of oropharyngeal dysphagia (OPD) (Da Costa et al., 2019; Krüger et al., 2017, 2019; Martinez-Biarge et al., 2012).

Injuries to areas such as the basal ganglia, thalamus and brainstem are frequently reported in neonates with HIE and can disrupt oral-motor function (Martinez-Biarge et al., 2012; Quattrocchi et al., 2010), leading to oral phase dysphagia (Krüger et al., 2017, 2019; Malan et al., 2022; Martinez-Biarge et al., 2012; Quattrocchi et al., 2010). As a result, neonates with HIE often present with shallow latching, poor suction, single sucks, short sucking bursts and reduced feeding endurance (Krüger et al., 2017, 2019; Malan et al., 2022), which may delay the attainment of full oral feeding, increase aspiration risk and prolong hospitalisation (Arvedson et al., 2020; Jadcherla, 2016). In addition, a disrupted pharyngeal swallow, particularly affecting suck–swallow–breathe (SSB) coordination and the initiation of an effective swallow reflex, may be observed (Malan et al., 2022), further increasing the risk for desaturation, bradycardia (Thoyre & Carlson, 2003) and aspiration (Viswanathan & Jadcherla, 2020).

Clinical signs such as coughing, choking and wet respiration have been observed in neonates with HIE and suggested laryngeal penetration or aspiration (Krüger et al., 2019). Importantly, Malan et al. (2022) found that 31% of neonates with HIE showed penetration or aspiration on the videofluoroscopic swallow study (VFSS), with 60% of the aspiration events occurring silently. While some improvement of swallowing may occur with maturation, many neonates with HIE remain at risk for persistent feeding difficulties at discharge (Jensen et al., 2017; Malan et al., 2022), potentially requiring long-term support and monitoring (Martinez-Biarge et al., 2012).

Evidence-based early intervention may assist in reducing the negative sequelae associated with OPD (Jadcherla, 2016), such as aspiration-associated respiratory disease (Durvasula et al., 2014), interrupted feeding interactions, psychological distress symptoms in the caregiver (Jonsdottir et al., 2021; Zanardo et al., 2017) and the length of hospitalisation (American Academy of Pediatrics, 2008).

There is a small but growing body of local research on neonates with HIE. Considering the prevalence of HIE in SA and the negative consequences associated with OPD, more dysphagia-specific research is justified to provide context-specific feeding and swallowing characteristics to further guide neonatal intervention.

The aim of this study was to describe the feeding and swallowing profile of neonates with HIE in a neonatal unit in Cape Town, South Africa, including characteristics of oral feeding readiness, time taken to reach full oral feeds, feeding method at discharge from the neonatal unit and clinical signs and symptoms of OPD.

## Methods

### Study design

A descriptive, exploratory, longitudinal study consisting of both prospective and retrospective data collection methods examined the feeding and swallowing characteristics in a sample of 52 participants with HIE of varying severities over the course of their hospitalisation or until the establishment of full oral feeds.

### Setting

The study was conducted at a regional public maternity and neonatal hospital in Cape Town, which is a referral facility for neonates with HIE.

### Study population and sampling strategy

Fifty-two participants were included in the study, 13 from prospective data collection and 39 from retrospective review of medical records. Neonates or medical records of neonates were included in the study if they met the following criteria: a primary diagnosis of HIE according to hospital protocol by the treating medical doctor, born ≥ 37 weeks of gestation and without comorbid conditions associated with OPD. They were classified according to the severity of their encephalopathy, as determined by their initial amplitude-integrated electroencephalography (aEEG) reading and whether or not therapeutic hypothermia was initiated (Table 1). The aEEG background is classified according to voltage changes on cerebral functioning monitoring, and an initial abnormal aEEG pattern is used to determine eligibility for hypothermia treatment, which should be initiated within the first 6 h of life (Gillam-Krakauer & Gowen, 2022). Participants were deemed medically stable by their treating doctor for a continuous period of 24 h prior to conducting the oral feeding assessments.

**TABLE 1:** Hypoxic ischemic encephalopathy categorisation of participants.

Participant groups	*n*
Total	52
Normal initial aEEG; not cooled	7
Severely abnormal initial aEEG; not cooled	6
Abnormal initial aEEG; cooled	26
Severely abnormal initial aEEG; cooled	13

*Source:* Adapted from Branfield, S. (2024). *Feeding and swallowing in neonates with Hypoxic Ischemic Encephalopathy (HIE): A descriptive study*. Master’s dissertation. University of Cape Town. http://hdl.handle.net/11427/40800

aEEG, amplitude-integrated electroencephalography.

### Data collection

Data were collected by the researcher (first author, S.B.), a qualified and registered speech-language therapist (SLT), as part of a postgraduate research project.

#### Prospective data collection

After obtaining informed consent, relevant case history information, such as biographical, birth, medical and feeding history, was obtained from the medical records, caregivers and treating clinicians using a standardised ‘Prospective Data Collection Form’. Feeding observations were systematically conducted at the bedside during typical feeding times. The Neonatal Feeding Assessment Scale (NFAS), an observation tool specifically developed and validated in South Africa (Viviers et al., 2016) to describe neonatal feeding and swallowing, was used. The NFAS includes six broad sections assessed, including physiological functioning, state of alertness during feeding, stress cues during feeding, general movement and muscle tone at rest and during feeding, an oral peripheral examination and a clinical feeding and swallowing evaluation (Viviers et al., 2016). All neonates in the unit are monitored with pulse oximeters, thereby allowing the researcher to document physiological parameters such as heart rate and oxygen saturation levels before, during and after feeding evaluations. Feeding and swallowing characteristics using the NFAS were recorded two to three times weekly until full oral feeding was achieved. The number of assessments varied depending on the time taken to reach full oral feeds with some participants having a single assessment (*n* = 7), while others required multiple observations (*n* = 6).

#### Retrospective data collection

The medical records of 39 neonates who met the inclusion criteria, out of a potential 71, were reviewed to obtain relevant case history information and feeding-related data. Detailed swallowing assessment information was only available for six of these participants who had been referred to and assessed by the resident SLT.

#### Validity and reliability

The NFAS was developed and validated in SA and was therefore contextually appropriate for use in this study. The NFAS demonstrated content, criterion and construct validity, as well as reliability during its validation (Viviers et al., 2019). In this study, a second qualified and registered SLT, also conducting research at the site, assisted in determining inter-rater reliability during both prospective and retrospective data collection with 100% agreement.

### Data analysis

Case history data were compiled into a single Excel spreadsheet, allowing for participants to be categorised according to encephalopathy severity (i.e. aEEG pattern) and whether therapeutic hypothermia was initiated.

Data regarding oral feeding readiness, time to full oral feeds and discharge feeding methods were analysed for all 52 participants. The oropharyngeal swallowing characteristics as assessed by the researcher using the NFAS and/or documented in the medical notes by the resident SLT were combined, organised and summarised.

Data were analysed using descriptive and inferential statistical methods to examine feeding and swallowing patterns in neonates with HIE. Descriptive statistics, including mean, range, median, interquartile range (IQR) and standard deviation, were used to summarise data. Inferential statistical tests, including the Kruskal-Wallis test and Mann-Whitney *U* test, were conducted to determine between-group differences in feeding outcomes, particularly in the time taken (days) to oral feeding readiness and time (days) to full oral feeds. Frequency counts and cross-tabulations provided insights into feeding and swallowing characteristics. A *p*-value of < 0.05 was considered statistically significant. Statistical analyses were conducted using SPSS (version 30) software to ensure accuracy and reliability (IBM Corp., 2024).

### Ethical considerations

Ethics approval was obtained from the University of Cape Town, Faculty of Health Sciences’ Human Research Ethics Committee on 10 December 2021 (approval number: HREC 780/2021) and renewed annually. Caregivers gave informed, voluntary, written consent in their preferred language, and infection control measures were followed throughout. Confidentiality was maintained by anonymising data, securing storage and offering private consultations when needed.

## Results

The results are presented according to the objectives of the study, to describe characteristics of oral feeding readiness, time taken to reach full oral feeds, feeding method at discharge from the neonatal unit and clinical signs and symptoms of OPD.

### Time to oral feeding readiness

The overall median time to achieve oral feeding readiness was 4 days (IQR: 4–5; s.d.: 3.7) with a statistically significant difference across groups (*p* = 0.036). Participants with a severely abnormal aEEG and who were not cooled (*n* = 4) took the longest to reach oral feeding readiness with a median of 12.5 days (IQR: 4.3–19.3; s.d.: 7.9), significantly longer than those with a normal aEEG (*p* = 0.042). No other statistically significant differences were observed between groups.

### Time taken to reach full oral feeds

The median time taken for participants to establish full oral feeds was 5 days with an IQR of 4–6.8 days and a standard deviation of 4.3 days (*N* = 50). Participants with a normal initial aEEG experienced the shortest time to full oral feeds (median = 1 day), whereas those with a severely abnormal aEEG and who were not cooled experienced the longest duration to full oral feeds (median = 13.5 days). These group differences are summarised in Table 2.

**TABLE 2:** Time taken to full oral feeds.

Participants	*N*	Median (days)	IQR (days)	s.d. (days)	*p*
Overall	50*	5.0	4–6.8	4.3	0.016
Normal aEEG; not cooled	7	1.0	16	5.6	-
Severely abnormal aEEG; not cooled	4	13.5	6.3–21.5	7.9	-
Moderately abnormal aEEG; cooled	26	5.0	4–6	3.6	-
Severely abnormal aEEG; cooled	13	6.0	5–7	1.5	-

*Source*: Branfield, S. (2024). *Feeding and swallowing in neonates with Hypoxic Ischemic Encephalopathy (HIE): A descriptive study*. Master’s dissertation. University of Cape Town. http://hdl.handle.net/11427/40800

IQR, interquartile range; aEEG, amplitude-integrated electroencephalography; s.d., standard deviation.

* , *N* = 50 (i.e. 52–2): Two participants with severely abnormal aEEG and not cooled were fed via an NGT at discharge.

Participants with a severely abnormal initial aEEG and who did not receive hypothermia treatment took significantly longer to establish full oral feeds compared with both the normal aEEG group (*p* = 0.042) and the moderately abnormal aEEG group (*p* = 0.022). No other statistically significant group differences were found.

### Feeding methods until discharge

Upon admission, all but one of the participants (98.1%; *n* = 51) received enteral feeding *via* nasogastric (NGT) or orogastric (OGT) tubes. On the first day of oral feeding trials, 44.2% (*n* = 23) of participants met their full intake requirements orally (i.e. breastfeeding/cup feeding). At discharge, 98% (*n* = 50) of the sample were receiving their full intake requirements orally, while the remaining two participants required long-term non-oral feeds.

### Clinical signs and symptoms of oropharyngeal dysphagia

Detailed information on swallowing characteristics was available for 19 participants through NFAS results or SLT assessment results in the medical notes. The oral feeding assessment indicated that OPD was likely to be present in 63.2% (*n* = 12) of the 19 participants (Table 3).

**TABLE 3:** Diagnostic outcome of oropharyngeal dysphagia in the oral feeding assessment.

Diagnostic outcome (NFAS; SLT assessment)	Overall *N* = 19			Not cooled				Cooled			
				Normal aEEG *n* = 2		Severely abnormal *n* = 6		Moderately abnormal *n* = 6		Severely abnormal *n* = 5	
	*N*	%	*p*	*n*	%	*n*	%	*n*	%	*n*	%
OPD likely to be present	12	63.2	0.053	2	100.0	3	50.0	3	50.0	4	80.0
Full NGT feeds recommended	3	23.1	-	0	0.0	2	33.3	1	16.7	0	0.0

Note: Please see the full reference list of this article, https://doi.org/10.4102/sajcd.v72i2.1121, for more information.

*Source*: Branfield, S. (2024). *Feeding and swallowing in neonates with Hypoxic Ischemic Encephalopathy (HIE): A descriptive study*. Master’s dissertation. University of Cape Town. http://hdl.handle.net/11427/40800

NFAS, Neonatal Feeding Assessment Scale (Viviers et al., 2016); SLT, speech-language therapist; OPD, oropharyngeal dysphagia; NGT, nasogastric tube; aEEG, amplitude-integrated electroencephalography.

**FIGURE 1:**
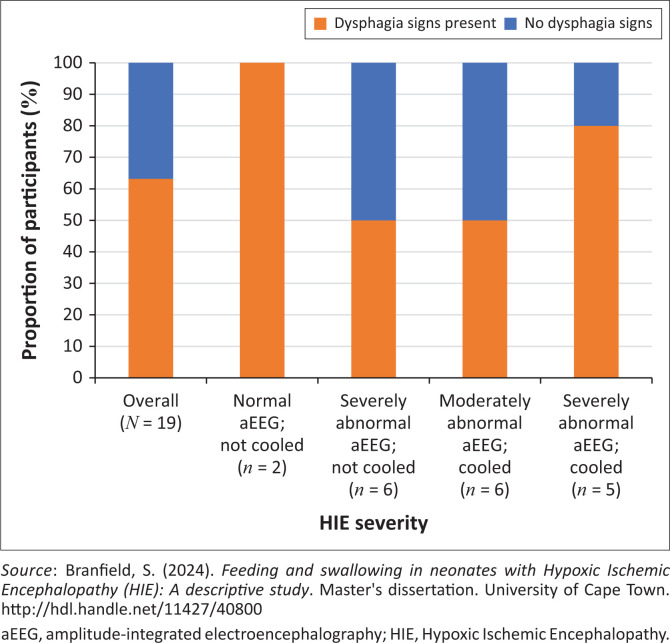
Proportion of participants who presented with clinical signs and symptoms of dysphagia in the oral feeding assessment (*N* = 19).

The oral feeding assessment revealed that participants across all aEEG severity groups were likely to present with OPD although group differences were not statistically significant (*p* = 0.053). The proportion of participants who were likely to present with OPD at the first oral feeding assessment is illustrated in Figure 1.

### Subsystem functioning during the initial Neonatal Feeding Assessment Scale assessment

The physiological stability, level of alertness and stress cues displayed by participants during the first NFAS assessment and the interaction between the subsystems provided additional oral feeding readiness information about the participants. Subsystem functioning was captured for 13 participants. All were medically stable per study criteria. Four (30.8%) participants exhibited autonomic instability (tachycardia or abnormal respiratory patterns) during feeding, suggesting that they may not have been ready for oral feeding as 3 (75.0%) presented with a diagnosis of OPD. Seven of the 13 participants (53.8%) demonstrated a non-optimal state of alertness during feeding, and of these, 4 (57.1%) presented with a diagnosis of OPD. All participants who presented with three or more stress cues during feeding and ≥ 2 disorganised physiological subsystems presented with a diagnosis of OPD (*n* = 4). None of the participants with an optimal state of alertness or less than three stress cues presented with a diagnostic outcome of OPD (Table 4).

**TABLE 4:** Physiological, behavioural and self-regulation sub-system functioning during the first Neonatal Feeding Assessment Scale and the diagnostic outcome of oropharyngeal dysphagia.

Sub-system	Response	Overall *N* = 13		
		Total *n*	OPD *n*	%
Physiological	Normal heart rate/respiration	9	1	11.1
	Tachycardia/abnormal respiratory patterns	4	3	75.0
Behavioural	Optimal state of alertness	6	0	0.0
	Non-optimal state of alertness	7	4	57.1
Self-regulation	< 3 stress cues	9	0	0.0
	≥ 3 stress cues	4	4	100.0
Subsystem interactions	Adequate^a^	9	0	0.0
	Inadequate^b^	4	4	100.0

*Source*: Branfield, S. (2024). *Feeding and swallowing in neonates with Hypoxic Ischemic Encephalopathy (HIE): A descriptive study*. Master’s dissertation. University of Cape Town. http://hdl.handle.net/11427/40800

OPD, oropharyngeal dysphagia.

a , Adequate: ≤ 1 unstable/disorganised subsystem (physiological/behavioural/self-regulation).

b , Inadequate: ≥ 2 unstable/disorganised subsystems (physiological, behavioural and/or self-regulation).

### Swallowing assessment

Participants were assessed during breastfeeding (78.9%; *n* = 15), cup feeding (11.1%; *n* = 1), breastfeeding supplemented with cup (11.1%; *n* = 1) and syringe feeding (10.5%; *n* = 2).

### Oral phase of swallowing

Clinical signs of OPD within the oral phase included inadequate lip closure/seal on nipple (26.3%; *n* = 5), reduced sucks per burst (52.6%; *n* = 10), delayed sucking responses (36.8%; *n* = 7), reduced sucking strength (42.1%; *n* = 8) and poorly coordinated SSB sequences (21.1%; *n* = 5). The clinical symptoms of OPD included pooling of saliva/secretions (10.5%; *n* = 2), pooling of the bolus (10.5%; *n* = 2), excessive anterior spillage during cup/syringe feeding (10.5%; *n* = 2) and reduced feeding endurance (63.2%; *n* = 12). The results from this subsection indicated that the majority of participants across all HIE severities presented with difficulties in the oral phase of swallowing (78.9%; *n* = 15) with reduced endurance and a reduced number of sucks per burst being the most common oral phase difficulties. The findings are summarised in Table 5.

**TABLE 5:** Clinical signs and symptoms of oropharyngeal dysphagia in the oral phase of swallowing.

Oral phase signs and symptoms (NFAS; SLT clinical notes)	Overall *N* = 19		Not cooled				Cooled			
			Normal aEEG *n* = 2		Severely abnormal aEEG *n* = 6		Moderately abnormal aEEG *n* = 6		Severely abnormal aEEG *n* = 5	
	*N*	%	*n*	%	*n*	%	*n*	%	*n*	%
**Clinical signs**										
Inadequate lip closure/seal on nipple	5	26.3	2	100.0	1	16.7	0	0.0	2	40.0
n/a (CF/SF)	3	15.8	0	0.0	2	33.3	1	16.7	0	0.0
< 10 sucks per burst cycle	10	52.6	2	100.0	3	50.0	1	16.7	4	80.0
n/a (CF)	1	5.3	0	0.0	1	16.7	0	0.0	0	0.0
Delayed initiation of NS	7	36.8	2	100.0	1	16.7	2	33.3	2	40.0
NR	1	5.3	0	0.0	1	16.7	0	0.0	0	0.0
n/a (CF)	1	5.3	0	0.0	1	16.7	0	0.0	0	0.0
Weak/poor NS response	8	42.1	1	50.0	3	50.0	1	16.7	3	60.0
n/a (CF)	1	5.3	0	0.0	1	16.7	0	0.0	0	0.0
Absent NS response	0	0.0	0	0.0	0	0.0	0	0.0	0	0.0
Uncoordinated SSB rhythm	5	21.1	0	0.0	2	33.3	2	16.7	1	20.0
**Clinical symptoms**										
Pooling of saliva/secretions	2	10.5	0	0.0	1	16.7	1	16.7	0	0.0
Pooling of the bolus	2	10.5	0	0.0	2	33.3	0	0.0	0	0.0
Inadequate lip closure with excessive anterior spillage (BF)	0	0.0	0	0.0	0	0.0	0	0.0	0	0.0
Excessive anterior spillage (CF/SF)	2	10.5	1	50.0	1	16.7	0	0.0	0	0.0
Reduced endurance	12	63.2	2	100.0	3	50.0	4	66.7	3	60.0
NR	1	5.3	0	0.0	1	16.7	0	0.0	0	0.0

**Total *N/n***	**15**	**78.9**	**2**	**100.0**	**4**	**66.7**	**5**	**83.3**	**4**	**80.0**

*Source*: Branfield, S. (2024). *Feeding and swallowing in neonates with Hypoxic Ischemic Encephalopathy (HIE): A descriptive study*. Master’s dissertation. University of Cape Town. http://hdl.handle.net/11427/40800

NFAS, Neonatal feeding assessment; SLT, speech-language therapist; BF, breastfeeding; CF, cup feeding; SF, syringe feeding; NR, not reported; NS, nutritive sucking; SSB, suck-swallow-breathe; n/a, not applicable; aEEG, amplitude-integrated electroencephalography

### The pharyngeal phase

Clinical signs of OPD included delayed (10.5%; *n* = 2) and absent (5.3%; *n* = 1) pharyngeal swallow responses. Observed clinical symptoms of OPD comprised gurgling (10.5%; *n* = 2), coughing (5.3%; *n* = 1) and teary eyes (5.3%; *n* = 1) during or immediately after the swallow. Pharyngeal phase difficulties were identified in 26.3% (*n* = 5) of participants during the oral feeding assessment.

## Discussion

This study found that while most neonates with HIE were deemed medically stable for oral feeding by the fourth day of life, their physiological stability, behavioural state organisation and their ability to self-regulate during feeding were influential in determining their oral feeding success during the initial feeding assessment. Oral feeding is a complex developmental process that requires stable physiological, motor and behavioural functioning (Viswanathan & Jadcherla, 2020). The findings reinforce the importance of a cue-based, behavioural approach to assessing oral feeding readiness. Recognising and responding to self-regulation cues allows caregivers and healthcare professionals to support neonates in achieving feeding success safely while also fostering a more positive and responsive feeding experience (Gulati et al., 2020).

Physiological instability – such as tachycardia, abnormal respiratory patterns and uncoordinated SSB rhythms – was common among participants with feeding and swallowing difficulties in this study, likely indicating a lack of readiness for oral feeding (Wahyuni et al., 2022). These findings align with literature linking autonomic instability to impaired feeding and swallowing function, possibly because of delayed skill development (Viswanathan & Jadcherla, 2020) or inappropriately timed oral feeding (Ross & Philbin, 2011). Similarly, difficulties in behavioural state organisation, particularly in neonates with non-optimal alertness (e.g. drowsiness, agitation), were observed among the participants with OPD (*n* = 4; 57.1%). These behavioural states indicate difficulties with state regulation, likely because of neurological impairment (Volpe, 2012) or the effects of medication (Mutanana et al., 2020). Notably, none of the neonates in optimal alert states were diagnosed with OPD, reinforcing the importance of behavioural readiness for safe and effective oral feeding (Gulati et al., 2020).

All neonates showing stress cues and poor self-regulation were diagnosed with OPD, suggesting that the feeding situation exceeded their capacity to self-regulate (Maltese et al., 2017; Wahyuni et al., 2022), thus underscoring the critical role of the self-regulatory subsystem in feeding success. Instability during feeding can lead to fatigue, poor endurance, disorganised behaviour, motor instability and ultimately unsafe and inadequate oral intake leading to a prolonged period of enteral feeding and hospitalisation (Arvedson et al., 2020). These findings suggest that instability across these interrelated subsystems compromises oral feeding readiness and success, emphasising the need for clinicians to observe behavioural and physiological cues when assessing oral feeding readiness to reduce aspiration risk and support better feeding outcomes.

The study highlights the variability in feeding among neonates with HIE with 44.2% (*n* = 23) requiring supplemental enteral feeds and 11.5% (*n* = 6) full enteral support at the initiation of oral feeding attempts – largely because of signs of oral phase dysfunction. Despite early challenges, most participants (96.2%; *n* = 50) achieved full oral feeds within a median of 5 days, likely because of neurological recovery (Medoff-Cooper et al., 2009), improved endurance (Lau & Smith, 2011), improved subsystem functioning and interaction (Jones, 2012) and intervention by the resident SLT for some participants.

Feeding outcomes such as oral feeding readiness, need for enteral support, time to full oral feeds and discharge feeding methods varied according to HIE severity, as indicated by aEEG patterns and whether or not therapeutic hypothermia was initiated. Neonates with a normal initial aEEG, indicating mild HIE, showed early oral feeding readiness and full oral feeding within 1 day, despite some signs of OPD. The participants who underwent therapeutic hypothermia treatment – those with moderately and severely abnormal initial aEEG – presented similarly with respect to the time taken to oral feeding readiness (4 and 5 days, respectively) and the time taken to full oral feeds (5 and 6 days, respectively). However, those with severely abnormal aEEG patterns who did not receive therapeutic hypothermia experienced the longest median time to oral feeding readiness (12.5 days) and the longest median time to the establishment of full oral feeds (13.5 days) with two participants requiring gastrostomy placement at the time of discharge.

Therapeutic hypothermia appeared to mitigate feeding difficulties in neonates with moderate to severe HIE, likely contributing to better feeding outcomes. These findings, while limited, support the neuroprotective role of therapeutic hypothermia (Bonifacio et al., 2022). Studies with larger sample sizes across multiple centres will, however, enable more accurate statistical comparisons of aEEG patterns, therapeutic hypothermia treatment and feeding and swallowing outcomes.

This study confirmed that neonates with HIE – regardless of severity – are at increased risk for feeding and swallowing difficulties, reinforcing HIE as a significant risk factor for OPD and its associated complications (Gulati et al., 2015; Jadcherla, 2016; Krüger et al., 2017, 2019; Malan et al., 2022). Oral phase signs and symptoms of dysphagia were observed in the majority (78.9%; *n* = 15) of participants in this study, though fewer (63.2%; *n* = 12) met the diagnostic criteria for OPD, possibly because of timing differences in assessments allowing for neurological recovery and skill development (Shandley et al., 2021). The most frequently reported oral feeding difficulties were short sucking bursts and reduced endurance, both of which are consistent with prior findings in neonates with HIE (Krüger et al., 2017, 2019; Malan et al., 2022). These challenges are linked to swallowing difficulties (Arora et al., 2022), fatigue and disorganised behavioural states (Vandenberg, 2007), potentially increasing the risk for aspiration, poor oral intake and prolonged enteral feeding and hospitalisation (American Academy of Pediatrics, 2008).

Weak sucking responses (42.1%; *n* = 8) and inadequate lip closure during breastfeeding also emerged as difficulties, suggesting latching and milk extraction problems (Lau, 2015), possibly because of abnormal muscle tone (Arvedson et al., 2020) associated with neurological impairment (Da Costa et al., 2019; Straathof et al., 2022). Although anterior spillage was not observed, despite reports of inadequate lip closure (26.3%; *n* = 5) during breastfeeding, weak sucking responses (42.1%; *n* = 8) likely resulted in an insufficient bolus size and therefore an insufficient bolus mass for an ‘excessive’ loss of liquid. Poor tongue cupping/grooving and anterior–posterior movement (52.6%; *n* = 10), which are crucial for effective bolus control and safe swallow initiation, were also noted. These impairments increase the risk for anterior spillage, premature spillage, penetration, aspiration and suboptimal nutritional intake (Arvedson et al., 2020).

In neonates, the acts of latching, sucking and swallowing are regulated by central pattern generators in the brainstem (Viswanathan & Jadcherla, 2020). However, effective feeding involves a coordinated effort from multiple brain regions. As such, hypoxic-ischemic injuries, which may affect several neural structures, can significantly disrupt these feeding functions (Jadcherla, 2016; Krüger et al., 2017, 2019; Malan et al., 2022; Martinez-Biarge et al., 2012; Quattrocchi et al., 2010; Shandley et al., 2021). The findings of this study – including poorly coordinated SSB sequences and other oral phase difficulties – likely reflect the impact of neurological injury commonly seen in HIE.

As hypoxic-ischemic injuries do not affect all brain structures at the same time and in the same way, brain regions with higher energy demands, such as the brainstem tegmentum (Miller et al., 2005), basal ganglia and thalamus (Martinez-Biarge et al., 2012; Miller et al., 2005; Quattrocchi et al., 2010), are particularly vulnerable to hypoxic-ischemic insults (Huang & Castillo, 2008) and play critical roles in feeding coordination. Damage to these areas, specifically the brainstem, has been strongly associated with oral-motor dysfunction in neonates with HIE (Quattrocchi et al., 2010). As the basal ganglia modulate the final motor output *via* the thalamus (Mistry & Hamdy, 2008), an impairment in the organisation and execution of the oral phase of swallowing in neonates with HIE has been associated with basal ganglia, thalamic and mesencephalic lesions (Martinez-Biarge et al., 2012), which could explain the oral phase difficulties observed in the present study.

Hypoxic ischemic encephalopathy may also adversely affect the pharyngeal phase of swallowing (Gulati et al., 2015; Jensen et al., 2017), though this cannot be confirmed clinically because of limitations in bedside assessments (Cordier et al., 2023). In this study, 26.3% (*n* = 5) of participants demonstrated clinical signs of pharyngeal dysphagia, but the absence of instrumental evaluations such as VFSS may have led to under-identification. Given the high number of oral phase difficulties and previous findings of abnormal pharyngoesophageal reflexes in neonates with HIE (Gulati et al., 2015; Jensen et al., 2017; Malan et al., 2022), it is likely that more participants presented with undetected pharyngeal phase impairments.

Only 10.5% of neonates were suspected of delayed pharyngeal swallows based on the clinical evaluation, whereas prior studies found higher prevalence rates on the VFSS than were identified on the NFAS (Malan et al., 2022). Delayed pharyngeal swallows can result in pooling of the milk bolus and increase aspiration risk (Jadcherla, 2016; Viswanathan & Jadcherla, 2020). Clinical signs of aspiration such as gurgling (10.5%; *n* = 2) and coughing (5.3%; *n* = 1) were rare. However, silent aspiration appears to occur more frequently in neonates and children who are neurologically compromised, making clinical inference more difficult (Freitag et al., 2021; Malan et al., 2022). Research indicates that impaired laryngeal sensation and reduced swallow reflex sensitivity in HIE may contribute to this silent risk (Freitag et al., 2021; Jensen et al., 2017); however, this relationship requires further investigation.

Overall, the study reaffirms that neonates with HIE commonly present with oral phase difficulties and possibly under-recognised pharyngeal phase impairments reinforcing the need for instrumental evaluations as part of routine care where clinically indicated. These findings are consistent with previous research showing that feeding and swallowing difficulties in HIE can span across the phases of swallowing (Gulati et al., 2015; Jensen et al., 2017; Krüger et al., 2017, 2019; Malan et al., 2022).

### Clinical implications

Neonates with HIE, regardless of severity, are at an increased risk for feeding and swallowing difficulties and should be screened for feeding difficulties and referred to SLTs once medically stable. Given South Africa’s high HIE incidence, dysphagia services should be accessible in all paediatric facilities. As neonates with HIE are at an increased risk for silent aspiration particularly, an instrumental assessment of swallowing – such as VFSS and fiberoptic endoscopic evaluation of swallowing (FEES) – may be required for the accurate diagnosis of aspiration and subsequent management (Malan et al., 2022). However, because of limited access to instrumental assessments, clinicians must remain vigilant and collaborate with specialised centres. Considering the increased risk for neurodevelopmental consequences such as cerebral palsy (Kukka et al., 2022), long-term follow-up of infants with HIE is recommended to ensure early intervention, reduce adverse outcomes and inform training for SLTs and healthcare professionals. This research informs current clinical practice and may contribute to education and training in the field of paediatric dysphagia.

### Limitations

This study has several limitations. While aEEG is an established prognostic tool for selecting neonates for therapeutic hypothermia (Horn et al., 2013), its application in dysphagia research is novel, limiting comparisons with studies using traditional HIE classifications. Additionally, while the NFAS is a valid tool for the South African context, it is limited in assessing the pharyngeal phase of swallowing, which cannot be fully explored. The tool may also not fully reflect clinical practice, as a diagnosis of OPD required multiple positive indicators within the assessment, potentially under-identifying difficulties. The retrospective nature of part of the study introduced inconsistencies and missing data with feeding difficulties likely under-reported because of limited SLT staffing and referral practices. Finally, the small sample with unequal subgroups and the use of a novel HIE classification limit generalisability and comparison with existing literature. Nonetheless, emerging patterns in feeding development offer valuable direction for future research.

## Conclusion

This study provided a detailed overview of the feeding and swallowing difficulties experienced by neonates with HIE in a referral hospital in Cape Town, South Africa, using a validated clinical assessment tool. The findings emphasise that oral feeding readiness is a dynamic and multifactorial process requiring continuous assessment until full oral feeding is consistently achieved. While the oral phase of swallowing appeared most affected, this could be because of the assessment tool’s limited ability to evaluate the pharyngeal phase. The results align with existing literature on motor outcomes associated with neurological injuries and highlight the increased risk of aspiration, prolonged enteral feeding, extended hospital stays and disrupted feeding interactions in this population. Given the high incidence of HIE in South Africa and its associated healthcare costs, the study reinforces the critical role of SLTs in the early identification and management of neonatal feeding difficulties. Health professionals working with neonates should remain aware of the feeding challenges faced by neonates with HIE to optimise intervention and care strategies.
